# Effects of different treatment frequencies of electromagnetic stimulation for urinary incontinence in women: study protocol for a randomized controlled trial

**DOI:** 10.1186/s13063-024-08103-y

**Published:** 2024-04-26

**Authors:** Chunmei Chen, Jing Zhang, Hong Zhang, Haiyan Li, Jucheng Yu, Yao Pei, Yuan Fang

**Affiliations:** 1 Department of Women’s Health, School of Medicine, Chengdu Women’s and Children’s Central Hospital, The Affiliatedffiliatedffiliated Women’s and Children’s Hospital, University of Electronic Science and Technology of China, Chengdu, China; 2 Department of Women’s Health, Jinniu Maternity and Child Health Hospital of Chengdu, Chengdu, China; 3 Jiangsu Department of Science and Technology, Jiangsu Province Pelvic Floor Rehabilitation Engineering Technology Research Center, Zhenjiang, China; 4 Li Chiu Kong Family Sleep Assessment Unit, Department of Psychiatry, Faculty of Medicine, The Chinese University of Hong Kong, Hong Kong, Hong Kong SAR China; 5 State Key Laboratory of Emerging Infectious Diseases and Centre of Influenza Research, School of Public Health, LKS Faculty of Medicine, The University of Hong Kong, Hong Kong, Hong Kong SAR China

**Keywords:** Urinary incontinence, Pelvic floor muscle training, Electromagnetic stimulation, Randomized controlled trial

## Abstract

**Background:**

Urinary incontinence is highly prevalent in women while pelvic floor muscle training is recommended as the first-line therapy. However, the exact treatment regimen is poorly understood. Also, patients with pelvic floor muscle damage may have decreased muscle proprioception and cannot contract their muscles properly. Other conservative treatments including electromagnetic stimulation are suggested by several guidelines. Thus, the present study aims to compare the effectiveness of electromagnetic stimulation combined with pelvic floor muscle training as a conjunct treatment for urinary incontinence and different treatment frequencies will be investigated.

**Methods/design:**

This is a randomized, controlled clinical trial. We will include 165 patients with urinary incontinence from the outpatient center. Participants who meet the inclusion criteria will be randomly allocated to three groups: the pelvic floor muscle training group (active control group), the low-frequency electromagnetic stimulation group (group 1), and the high-frequency electromagnetic stimulation group (group 2). Both group 1 and group 2 will receive ten sessions of electromagnetic stimulation. Group 1 will be treated twice per week for 5 weeks while group 2 will receive 10 days of continuous treatment. The primary outcome is the change in International Consultation on Incontinence Questionnaire–Short Form cores after the ten sessions of the treatment, while the secondary outcomes include a 3-day bladder diary, pelvic floor muscle function, pelvic organ prolapse quantification, and quality of life assessed by SF-12. All the measurements will be assessed at baseline, after the intervention, and after 3 months of follow-up.

**Discussion:**

The present trial is designed to investigate the effects of a conjunct physiotherapy program for urinary incontinence in women. We hypothesize that this strategy is more effective than pelvic floor muscle training alone, and high-frequency electromagnetic stimulation will be superior to the low-frequency magnetic stimulation group.

**Supplementary Information:**

The online version contains supplementary material available at 10.1186/s13063-024-08103-y.

## Administrative information

Note: The numbers in curly brackets in this protocol refer to the SPIRIT checklist item numbers. The order of the items has been modified to group similar items (see https://www.equator-network.org/reporting-guidelines/spirit-2013-statement-defining-standard-protocol-items-for-clinical-trials/).

**Table:** 

Title [1]	Effects of different treatment frequencies of electromagnetic stimulation for urinary incontinence in women: study protocol for a randomized controlled trial
Trial registration {2a and 2b}	Chictr.org.cn, Chinese clinical trial registry, ID: ChiCTR2200061035
Protocol version {3}	Protocol version 2, 6 Oct 2022
Funding {4}	Funding for this study is from Chengdu Municipal Health Commission
Author details {5a}	Chunmei Chen^1^, Jing Zhang^1^, Hong Zhang^2^, Haiyan Li^3^, Jucheng Yu^4^, Yao Pei ^5^ and Yuan Fang^3^. ^1^Department of Women’s Health, Chengdu Women’s and Children’s Central Hospital, The affiliated Women’s and Children’s Hospital, School of Medicine, University of Electronic Science and Technology of China, China; ^2^Department of Women’s Health, Jinniu Maternity and Child Health Hospital of Chengdu, China; ^3^Jiangsu Province Pelvic Floor Rehabilitation Engineering Technology Research Center, Jiangsu Department of Science and Technology, China; ^4^Li Chiu Kong Family Sleep Assessment Unit, Department of Psychiatry, Faculty of Medicine, The Chinese University of Hong Kong, Hong Kong SAR, China; ^5^State Key Laboratory of Emerging Infectious Diseases and Centre of Influenza Research, School of Public Health, LKS Faculty of Medicine, The University of Hong Kong, Hong Kong SAR, China
Name and contact information for the trial sponsor {5b}	Chengdu municipal Health Commission, China. 609,996,578@qq.com
Role of sponsor {5c}	The sponsor played no role in the trial designing, data analysis and interpretation, and writing of the manuscript.

## Introduction

### Background and rationale {6a}

Urinary incontinence (UI) is a highly prevalent health issue globally. UI is described as involuntary leakage of urine, and it happens in both sexes but is more commonly seen in women [2]. The risk factors of UI in women are usually associated with impaired bladder and pelvic floor muscle function, and such conditions usually arise with pregnancy and menopause. The prevalence of UI gradually increases with aging. Around 6.9% of women between 20 and 39 years old suffer from UI, increasing to 31.7% in women aged above 80 [3, 4]. Based on the criteria of the International Continence Society (ICS) and the International Urogynecological Association (IUGA), there are two main subtypes of UI: urgency urinary incontinence (UUI) and stress urinary incontinence (SUI) [2]. SUI is usually associated with an increase in intra-abdominal pressure, while UUI is often associated with a sudden desire to void that is usually difficult to defer [5]. Mixed urinary incontinence (MUI) is a condition when the two subtypes coexist. Since UI could lead to limited physical activity, damaged sexual function, and other psychological problems, it is detrimental to the patient’s psychosocial well-being and quality of life [6].

Pelvic floor muscle training (PFMT) is regarded as the first-line therapy for UI in terms of a wide range of spectrums, including treatment response, quality of life (QoL), the volume of urine leakage, and treatment satisfaction [7]. PFMT could improve the stability of the urethra. A single bout of pelvic floor muscle contraction can narrow the levator hiatus area, increase the urethral closer pressure, and lift the rectum and bladder, thus reducing the symptoms of UI [8]. However, most of the comparisons with other active treatments were made in small, single-center trials, making it difficult to draw the conclusion of whether PFMT is superior to other treatments such as electromagnetic stimulation or vaginal cones [9]. Also, it is unclear whether combining PFMT with these treatments could have additional benefits.

Recent studies have attempted to investigate the different treatment strategies to maximize the efficacy of PFMT, including non-invasive electrical stimulation, biofeedback, and extracorporeal magnetic stimulation for lower urinary tract symptoms in women [10–13]. Electrical stimulation applies an intravaginal probe or surface electrodes that do not penetrate the skin, while magnetic stimulation utilizes a magnetic coil to generate an eddy current in the target tissue to trigger muscle contraction. Both of the two are passive muscle training. Biofeedback is defined as verbal or visual feedback to the participants to help with muscle contraction and relaxation, especially for patients with pelvic muscle damage who cannot contract them correctly [7]. These strategies are becoming important components of treatment modules and routine care for patients with UI. According to Chinese clinical settings, electrical stimulation and magnetic stimulation are usually combined as an adjunct treatment to assist PFMT. However, a consensus on the treatment regimen has not been achieved, such as treatment frequency.

### Objectives {7}

The first aim of the present study is to investigate the efficacy and safety of combining electromagnetic stimulation with PFMT for patients with UI. The second aim is to delineate the optimal treatment frequency of this strategy.

### Trial design {8}

This trial is a randomized controlled trial with a post-intervention follow-up of 3 months. Participants who meet the inclusion criteria will be included in the study. All the participants will be randomly allocated to the intervention and control groups in a 1:1:1 ratio using a randomization sequence.

## Methods: participants, interventions, and outcomes

### Study setting {9}

The three groups are the active control group: PFMT, twice per week for 5 weeks; group 1 (low-frequency magnetic stimulation group): PFMT + electromagnetic stimulation, twice per week for 5 weeks, every two sessions will be separated by at least 48 h; group 2 (high-frequency magnetic stimulation group): PFMT + electromagnetic stimulation, continuous treatment for 10 days. All the patients will also receive a 3-month post-intervention follow-up. The randomization process will be conducted by an online random number generator. The random numbers will be kept in opaque envelopes. Then, a research assistant will allocate the numbers to the participants according to the order of inclusion. The present study cannot blind the practitioner and the participants due to the nature of the interventions. However, the outcome assessor and data analyst will be blinded.

### Eligibility criteria [1]

The following are the inclusion criteria:

- Women aged 18–75 years.
- Women who report at least three episodes of involuntary urine leakage weekly during the preceding 3 months [14].
- Types of UI will be confirmed by the Questionnaire for Incontinence Diagnosis [15].

The following are the exclusion criteria:

- Body mass index ≥ 35 (equals weight in kilograms divided by height in meters squared)
- Restricted body movement
- Pelvic Organ Prolapse Quantification System >  stage 2
- History of pelvic surgery or physiotherapy
- Use of medication for UI or affects skeletal muscle function
- History of electromagnetic stimulation therapy for UI
- Neurological diseases
- Pregnancy
- Metal implants in the body, such as a pacemaker
- Other risk factors or comorbidities that interfere with the study

### Who will take informed consent? {26a}

Female patients aged from 18 to 75 who report weekly involuntary urine loss of three or more episodes for the past 3 months will be included, such criteria have been used widely in previous RCTs on UI [14]. Then, the validated Questionnaire for Incontinence Diagnosis (QUID) will be applied to confirm the type of UI as a pattern of stress/mixed UI. The eligible participants will be provided written informed consent with information illustrating the purpose and the detailed procedures of the present study after they meet the eligibility criteria. The consent will be obtained by the obstetricians and will be kept confidential and aimed only at research purposes. Data collected will be stored in a locker at the principal investigator’s office, and an electronic version will be protected by a password on the hospital computer.

### Additional consent provisions for collection and use of participant data and biological specimens {26b}

No biological specimens will be collected from this study. If additional data or biological specimens are required, they must be authorized by ethical boards and be included in the consent.

### Interventions

#### Rationale for the choice of comparators {6b}

The participants in the active control group will receive PFMT, which is recommended as the first-line therapy for UI [7]. Patients with UI are usually informed to do PFMT at home or under supervision by physiotherapists.

#### Intervention description {11a}

All the participants in the three groups will receive PFMT. The efficacy of PFMT has been reported widely in improving or curing UI in all age groups [16]. The International Continence Society published the guideline on managing incontinence and suggests that supervised PFMT should be considered as the first-line treatment for stress and mixed UI in women [17]. Due to the consideration of manpower and financial resources, the evidence from the GROUP study showed that the group-based PFMT can also demonstrate similar effects for the treatment of UI when compared with supervised individual PFMT [14]. To maximize the effect of PFMT and the patient’s adherence, the psychological self-determination theory (SDT) will be embedded in this treatment program [18]. SDT has been widely adopted in the literature on exercise behavior, and it assumes that all humans possess three psychological needs: a need for autonomy, a need for relatedness, and a need for competence [19]. Rationale of the PFMT will be provided for the patients, as well as clear goals with feedback and interpersonal interaction. All the abovementioned SDT strategies have no direct effect on UI.

Each electromagnetic stimulation session consists of 10-min electrical and 20-min magnetic stimulation. Electrical and magnetic stimulation devices (B4P electrical stimulator and MTS magnetic stimulator, Nanjing Medlander Medical Technology Co., Ltd.) will be applied for delivering the intervention. Details of the intervention are illustrated below (Table 1). All the uro-physiotherapists will be trained before the initiation of the study to ensure the delivery of a standardized treatment procedure. The standardized procedure includes the delivery of PFMT and the manipulation of the electrical and magnetic stimulation device.

**Table 1:** Standardization of training and protocols

Group	Standardized training program	Detailed dosage	Time in total
Active control group	Sustained contractions	Patients complete PFMT by themselves, 6–10 s for each contraction, and every two contractions are separated by a period of 10-s rest; 8–10 reps per set for 1–2 sets	50 min
	Phasic contractions	The patient completes pelvic floor muscle contractions of 2–5 s each, followed by rest intervals of 4–10 s. Each set contains 10 contractions, 1–3 sets each	
	Training guidance	Under the guidance of the instructor, patients perform PFMT, combined with breathing and relaxation training	
Group 1	PFMT + + electromagnetic stimulation	PFMT is the same as the active control group Electrical stimulation: 20–60 Hz, the pulse width of 200–600 μs, 10 min of stimulation Magnetic stimulation: 15 Hz for 2 s and 30 Hz for 3s, with a rest interval of 5 s, 20 min of intervention Twice per week for 5 weeks	
Group 2	PFMT + + electromagnetic stimulation	PFMT is the same as the active control group Electrical stimulation: 20–60 Hz, the pulse width of 200–600 μs, 10 min of stimulation Magnetic stimulation: 15 Hz for 2 s and 30 Hz for 3 s, with a rest interval of 5 s, 20 min of intervention 10 consecutive days	50min

### Active control group

Trained uro-physiotherapists will help the patients to initiate the PFMT program. At the beginning of the first session, the uro-physiotherapists will inform the patients of the history of PFMT and its benefits. Patients will be trained in a group of 6–8 patients. Before the training, clear goals for each session and the program as a whole will be given to all the patients. After each training session, the patient will fill out a treatment checklist and give suggestions related to the training. Uro-physiotherapists will then provide written/oral feedback for the patients on their improvement and concerns about their training. All the uro-physiotherapists will meet with the research team to ensure the consistency of the training protocol and discuss the concerns that may occur during the delivery of the training. The PFMT program meets the American College of Sports Medicine principles for prescribing exercise, including frequency, intensity, time, type, volume, and progression [20]. Patients in the active control group will receive PFMT twice per week for 5 weeks, making a total of 10 sessions. Every 2 sessions will be separated by at least 48 h. Each session lasts 50 min with a target of increasing pelvic floor muscle strength, power, endurance, and coordination in daily life context. The detailed training protocol will follow the Consensus on Exercise Reporting Template (CERT) [21]. It provides a direction for reporting exercise-related interventions regardless of exercise type and includes 16 items under 7 domains: what, who, how, where, when, dosage, tailoring, and compliance.

### Group 1 (low-frequency electromagnetic stimulation)

Both electrical and magnetic stimulation are widely applied by the Department of Obstetrics and Gynecology as well as pelvic floor rehabilitation clinics for incontinence treatment. All the treatment will take place in a private environment. For the electrical stimulation section, patients will be asked to take off their pants and then lie on the treatment couch in the lithotomy position. After disinfection and the application of lubricating gel, a J-shape electrode will be placed into the vagina by an experienced uro-physiotherapist. The electrode is connected to a computer that controls the output of the current. Group 1 patients will receive the treatments with a frequency of 20–60 Hz and a pulse width of 200–600 μs. At the same time, to ensure that the patients do not suffer from any pain from electrical stimulation, the treatment amplitude will be adjusted according to the patient’s tolerance to the stimulation. After 10 min of electrical stimulation, patients will be asked to sit on a magnetic stimulation chair. A magnetic coil that can generate a magnetic field will be placed within the treatment chair’s seat. The magnetic coil will be controlled by an external power unit by the same therapist that delivers electrical stimulation therapy. The output of the magnetic stimulation consists of 15 Hz for 2 s and 30 Hz for 3 s, with a rest interval of 5 s, making up a total treatment period of 20 min. The treatment frequency will be two sessions per week for 5 weeks; a total of ten sessions will be initiated. Every two sessions will be separated by at least 48 h. After the electromagnetic stimulation, patients will complete a 20-min PFMT session, which will be the same as the active control group.

### Group 2 (high-frequency electromagnetic stimulation)

Previous studies showed that low-frequency electromagnetic stimulation (twice or three times per week) has demonstrated efficacy in treating UI [22, 23]. However, one study showed that electrical stimulation was no more effective compared with sham treatment, which was controversial with other studies [24]. High-frequency electromagnetic stimulation refers to a regimen that uses a continuous daily treatment protocol, which has also demonstrated safety and efficacy in the treatment of UI [25, 26]. Another advantage of high-frequency electromagnetic stimulation is that patients can focus on the treatment and complete the entire course of treatment in a short period, with similar treatment effects compared with low-frequency electromagnetic stimulation. In the present study, we will adopt a 10-day, daily treatment regimen to match the total treatment volume with group 1. Each treatment session will be the same as group 1, to compare the effects of different treatment frequencies.

#### Criteria for discontinuing or modifying allocated interventions {11b}

Patients of the study are allowed to withdraw from the study at any time for any reason if they wish to do so without any consequences. The researchers of the study can also discontinue the participation if the participant is uncooperative and does not attend the study visits, and this patient will not be replaced by a new patient.

#### Strategies to improve adherence to interventions {11c}

To improve patient adherence, individuals who participate in the study will be closely supervised by interventionists with feedback for each treatment session. On an institutional level, all the interventionists and the researchers will have discussions on improving the delivery of the intervention and exchange their opinions on a weekly basis. Details of each intervention session will be recorded in a standardized document.

#### Relevant concomitant care permitted or prohibited during the trial {11d}

Participants who are taking medication for UI or have an impact on the skeletal muscle are prohibited from participating in the trial. Participants anticipated to wear a pacemaker due to a cardiac issue are also prohibited from enrollment. The research team will establish good palliative and emergency care management in case of any potential life-threatening issues.

#### Provisions for post-trial care {30}

The aim of the trial is to establish a comprehensive and standardized program for UI, and the participants will be able to continue beyond the trial. According to previous research, the trial does not include any specific risks, and there will be no provisions for compensation.

### Outcomes {12}

The primary outcome of the study is the International Consultation on Incontinence Questionnaire–Short Form (ICIQ-SF) [27]. It is a widely used four-item questionnaire with full validation that assesses the voiding frequency, voiding volume, impact on quality of life, and the cause of leakage during the past 4 weeks. The total score of the questionnaire ranges from 0 to 21, with 0–7 representing mild UI, 8–12 representing moderate UI, and 13–21 representing severe UI.

The secondary outcomes include (1) a 3-day bladder diary, which records the micturition frequency, incontinence episodes, fluid intake, times of voiding, voided volume, and pad usage per 24 h for 3 days. Previous observational studies have demonstrated the reliability and reproducibility of data from bladder diaries and standard symptom evaluation [28]. The duration of the diary between 3 and 7 days is usually reported in the literature, while the present study plans to apply the 3-day voiding diary due to the consideration of patients’ compliance and convenience. (2) Pelvic floor muscle function, which includes the modified Oxford Grading Scale, pelvic floor electromyography, and pressure measurements. With high inter- and intra-rater reliability [29, 30], the modified Oxford Grading Scale allows the quantification of pelvic floor muscle strength as 0, no contraction; 1, flicker; 2, weak; 3, moderate; 4, good; and 5, strong. The Glazer Protocol-embedded electromyography assesses the muscle activities during different stages such as baseline rest, phasic contraction, tonic contraction, endurance contraction, and post-contraction rest. The results of the test could reflect the supportive, sexual, and sphincter function of the pubococcygeus muscle [31]. Pressure measurement is another traditional way to assess pelvic floor muscle function, along with Oxford Grading Scale and electromyography; this multidimensional assessment model allows for a more comprehensive evaluation of pelvic muscle function. (3) Pelvic organ prolapse quantification (POP-Q) system, which is widely used in clinical settings and provides the characterization of a woman’s prolapse. The detailed technique of performing a POP-Q test has been described in the ICS/IUGA statements [2, 32]. (4) SF-12 Health Survey, which is one of the most widely used instruments that assess self-reported health-related quality of life. Compared to the original version SF-36 [33], the SF-12 covers all eight domains with fewer questions, which makes it more practical for patients to complete. Meanwhile, the Chinese version of SF-12 has been validated, which has similar psychometric properties as the English version [34].

#### Project timeline {13}

The timeline is presented in Table 2.

**Table 2:**
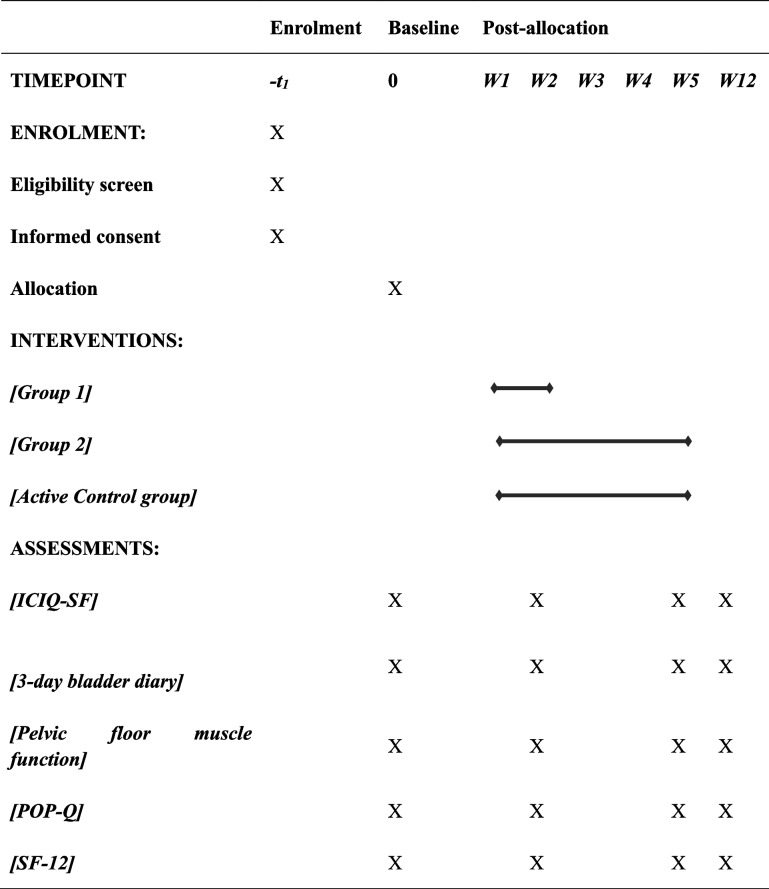
The participant timeline

#### Sample size {14}

The present study intends to apply ICIQ-SF as the primary outcome. After calculation, a sample size of 44 in each group is able to provide 80% power to detect a within-between-group difference measured using ICIQ-SF at three time points, assuming a small effect size (Cohen’s *f*) of 0.125 and a two-sided significance level of 5%. Considering that there is a 20% dropout rate, the study will increase the sample size to 165 in total.

#### Recruitment {15}

Female patients who report at least three episodes of involuntary urine leakage weekly during the preceding 3 months will be recruited. A poster will be posted on the bulletin board to illustrate the study details including the research background, criteria for enrollment, potential benefits of the intervention, and other rights of the participants. Patients who meet the inclusion criteria of the study will be given the information sheet, and researchers of the study will demonstrate the details of the study to the patients. Those who have an interest in joining the study will sign the consent forms.

### Assignment of interventions: allocation

#### Sequence generation {16a}

After confirming the consent, statistical program R will be applied to generate a randomization sequence. The allocation will be carried out by a research assistant who has no role in delivering the intervention and the data analysis. All the participants will be allocated in a 1:1:1 ratio.

#### Concealment mechanism {16b}

A circulating nurse who will not take part in this trial will be able to get access to the online randomization database. The investigator will be informed by the nurse that the participant is in the active control group or intervention group.

#### Implementation {16c}

After signing the informed consent forms, participants will be allocated to one of the study arms according to the sequence. The study group will also be revealed at the same time to the participants and the researchers of the study.

### Assignment of interventions: blinding

#### Who will be blinded {17a}

As the present trial is open-labeled, all the participants will not be blinded due to the major difference between the groups (frequencies of the treatment and whether electromagnetic stimulation will be delivered).

#### Procedure for unblinding if needed {17b}

The study does not include any unblinding procedure as this is an open-labeled trial. How about the outcome assessors? You can still name this study as single-blinded.

### Data collection and management

#### Plans for assessment and collection of outcomes {18a}

All the data will be collected at baseline, and how about at the end of the high-frequency group intervention? At the end of week 5 and 3 months after the intervention. A training diary will be given to all the individuals to record their treatment, including training frequency, total time of each meeting, parameters of each stimulation session, and the sum of the treatment time. The aim of this strategy is to ensure that the total volume of the treatment remains the same. The interventionist will record the treatment after each session, making sure that the PFMT and the electromagnetic stimulation protocols remain constant across the treatment. A validation study of the primary outcome, ICIQ-SF, shows that it is significantly related to the Patient Global Impression of Improvement and the 24-h pad test [35]. All the patients will be informed not to take bladder medicine for at least 2 weeks before completing the 3-day bladder diary. The patients will also be asked not to alter their habits of fluid intake and retain their voiding habits during the trial. The urine output will be measured using calibrated cups, and the recording will begin at 8 am and end at 7:59 am the next day [36]. These along with other secondary outcomes will be collected at baseline, at week 5, and after 3 months.

#### Plans to promote participant retention and complete follow-up {18b}

Before and during the intervention, participants will be given extensive information related to the importance of electromagnetic and PFMT for UI. Participants are allowed to discontinue at any time point of the study without giving a reason. However, if possible, these participants will also be asked to provide the outcomes of the study at week 5 and follow-up. Questionnaires, bladder diary, and pelvic floor muscle function are routinely applied in urology outpatient and are characterized by efficiency and convenience.

#### Data management {19}

An electronic case report form (eCRF) will be applied to record the data. Data of included participants will be entered into a web-based system Ennov Clinical® 7.5.720. Participants will be identified using the inclusion number, the first letter of their family name, and their given name. This eCRF will be kept by the coordination site of the study. All the data will be entered twice by two individual research assistants.

#### Confidentiality {27}

The full name of each participant and the identification code will be stored in a document kept by the principal investigator. Only the research team can get access to the key to the document. Participants’ private data will not be reported in the publication.

#### Plans for collection, laboratory evaluation, and storage of biological specimens for genetic or molecular analysis in this trial/future use {33}

No biological specimens will be collected in the trial. Questionnaires and electromyography data will be kept in the hospital’s online system as routine clinical assessment and may be used for future cohort studies.

## Statistical methods

### Statistical methods for primary and secondary outcomes {20a}

The main analysis will follow the intent-to-treat principle with all the randomized patients included. The present study will keep the missing data and will not replace them. Normally distributed variables will be presented as mean (SD) values. Non-normally distributed continuous variables will be reported as medium values with interquartile ranges (25th–75th percentile), and percentages will be used for categorical variables. The Shapiro–Wilk test will be used to examine the normal distribution of the variables.

Baseline demographic data will be reported for all patients randomized to the three groups. These data include patients’ age, education level, employment, annual household income, and medical history (BMI, ever pregnant, cigarette smoking, and diabetes). Baseline data will be evaluated by *χ* ^2^ or Fisher’s exact for categorical variables, analysis of variance (ANOVA) for continuous variables, and the Kruskal–Wallis test for non-parametric variables.

Generalized estimation equation (GEE) models will be applied to analyze primary and secondary outcomes collected at different time points. Considering the potential effects of age, BMI, severity of UI, and other variables on the outcomes, subgroup analysis will be carried out to evaluate whether age (< / ≥  50 years), the severity of UI (ICIQ-SF < </ ≥  13 points), and BMI (< / ≥ 25 kg/m^2^) at baseline could have an impact on the effect of the treatment. In view of the fact that patients may not be able to complete all courses of the treatment due to various reasons during the treatment, we will also conduct per protocol analysis. Patients who have completed the original intervention as described above will be included.

### Interim analyses {21b}

ICIQ-SF and a 3-day bladder diary will be collected at the end of week 2 as interim analysis to make potential modifications for the planned sample size based on the accumulating data within the trial.

### Methods for additional analyses (e.g., subgroup analyses) {20b}

No subgroup analysis was planned due to the small sample size of the study.

### Methods in analysis to handle protocol non-adherence and any statistical methods to handle missing data {20c}

The main analysis of the study will adopt the intention-to-treat (ITT) method, in which all the patients will be included in the final analysis according to the random allocation regardless of the study completion. Also, generalized estimation equation (GEE) models will be used to analyze the outcomes collected at different time points. This approach could fit the outcomes at different time points so all the participants are allowed in the analysis, even if missing data exists. The study will report the rate of missing data, and the missing data will not be replaced.

### Plans to give access to the full protocol, participant-level data, and statistical code {31c}

We will follow the requirements with regard to submitting the data to the journal where we publish the results of the study. After the publication, the full protocol, participant-level data, and statistical code will be available under reasonable request.

### Oversight and monitoring

#### Composition of the coordinating center and trial steering committee {5d}

The two authors from the coordinating center will take full responsibility for conducting the intervention, scientific validity, and patient management. A report of the progress will also be submitted to the ethics review board of the coordinating center on a monthly basis. The trial steering group will review and provide guidance on subject recruitment, evaluation, and treatment to ensure consistency in all operations. All the authors will participate in periodic meetings to discuss the progress of the study and strategies to better manage the trial weekly.

#### Composition of the data monitoring committee, its role, and reporting structure {21a}

The trial is going to be conducted in accordance with the Good Clinical Practices and Chinese regulations. The present study does not declare a data and safety monitoring board as electromagnetic stimulation and pelvic floor muscle training are both regarded as low risk and have been used routinely in patients who need medical care urgently.

#### Adverse event reporting and harms {22}

Any adverse events (AEs) will be reported to the study staff and the ethics committee of the Chengdu Women’s and Children’s Central Hospital. The AEs will be managed by the physician in charge of the participant’s care.

#### Frequency and plans for auditing trial conduct {23}

The audit of the trial will be performed weekly by a member designated by the sponsor of the study and will be independent of the study steering committee, the sponsor, and the investigators. All the members of the project management group will meet at least once every 2 weeks, whether online or face-to-face, to review the progress of the trial and ensure normal progress. Since it is considered as a low-risk intervention, we did not have a data monitoring committee.

#### Plans for communicating important protocol amendments to relevant parties (e.g., trial participants, ethical committees) {25}

Any revision to the trial will be reported to the ethics committee and the trial registry. After receiving the approval of the protocol revision, we will communicate it to the study participants, the sponsors, and the investigators.

#### Dissemination plans {31a}

The trial is registered on chictr.org.cn with the reference number ChiCTR2200061035. The results of the study will be published in a peer-reviewed medical journal. The investigator will also communicate the trial results to the national and international scientific community and healthcare providers at different conferences.

#### Patient public involvement

No patients or other public involvement in the design of the research protocol.

## Discussion

The first aim of this randomized controlled trial is to provide a comprehensive physiotherapy program that combines PFMT and electromagnetic stimulation adapted to the needs of women with UI. The anticipated results of this trial could be incorporated into routine clinical practice and offer an effective strategy to improve symptoms of UI and their general well-being. It will also recommend a suitable and standardized protocol that consists of different conservative interventions for patients with UI.

As previously mentioned, PFMT is recommended as the first-line therapy for patients with lower urinary tract symptoms [7]. However, a number of issues do exist when considering providing PFMT for UI patients. In China, it is difficult to provide one-on-one supervised PFMT for patients due to the heavy workload of healthcare providers and the number of patients. Compared with individualized PFMT, group-based PFMT, while having a similar effect [14], is still difficult to generalize to a large population. The primary reason is that it is difficult to make appointments with multiple patients at the same time in Chinese clinical settings, and the patient’s subjective perception and rehabilitation needs cannot be met. According to two Cochrane reviews, electrical stimulation could improve symptoms of lower urinary tract symptoms, such as overactive bladder and stress UI [37, 38]. However, previous studies should also be interpreted with caution since some of the studies were deemed to have a relatively high risk of bias including baseline differences between the groups and small sample sizes. Also, the mode and the type of delivery of such interventions remain controversial and poorly standardized (e.g., intervention frequency) as suggested by the European Association of Urology (EAU)’ guideline [39]. Based on the above research status, the present study is the first to explore the effectiveness of high-frequency versus low-frequency electromagnetic stimulation in the treatment of UI. Thus, one of the strengths of the present study is that the conjunct effect of PFMT and electromagnetic simulation can be evaluated with rigorous quality control, and a standardized intervention frequency can be established. Another strength of the study is that both patients’ subjective data and objective data will be collected for a comprehensive analysis of their symptoms and the impact on their quality of life.

There are several limitations of the current trial. Firstly, it is difficult to keep the participants blinded to the intervention due to the nature of electromagnetic stimulation and PFMT. The researchers will explain the details of the study carefully and inform the participants about the benefits of different interventions. Secondly, the age of the participants ranges from 18 to 75, which is heterogeneous and UI types should be different. We will consider subgroup analysis that may include different age groups and UI types when conducting the research.

## Trial status

The present study is in the recruiting phase, which started in July 2022 and will end in June 2023. The whole trial will be completed by the end of December 2023.

### Supplementary Information

**Supplementary Material 1.**

## Data Availability

The final de-identified dataset will be made available after publication of the study outcomes, if required by the journal publication policy or via a request to the corresponding author.

## Abbreviations

AEs: Adverse events
UI: Urinary incontinence
SUI: Stress urinary incontinence
PFMT: Pelvic floor muscle training
QoL: Quality of life
IUGA: International Urogynecological Association
ICS: International Continence Society
UUI: Urgency urinary incontinence
MUI: Mixed urinary incontinence
RCT: Randomized controlled trial
EAU: European Association of Urology
